# Supplementary material for: Obtention and preliminary clinical evaluation of an equine albumin for intravenous administration in adult Colombian Creole Horses (Equus ferus caballus)
Source: PLoS One. 2026 Feb 20;21(2):e0341577. doi: 10.1371/journal.pone.0341577 (PMC12922972; doi:10.1371/journal.pone.0341577)
Supplement: S1 File — Table S1. Original result data tablesheet (in spanish) obtained from the chromatography software.; Table S2. Transcribed and translated data sheet results table obtained from the software (the original is available in the supplementary material). Highlighted data indicates the percentage of relative abundance of the fraction corresponding to human albumin (HA).; Figures S1. Original sterility assay result issued by the GLP certified laboratory (in spanish) indicating the sterility of A: Batch AL-15052023. B: Batch AL-02042024. C: Batch AL-10042024. Figures S2: Original hemotropic assay result issued by the GLP certified laboratory (in spanish) indicating the absence of Anaplasma sp, Babesia sp, Mycoplasma sp, Trypanosoma sp and Theileria sp. on batch AL-15052023. Table S3. Table results with the physiological constants including the arterial preasure measurment. Table abreviations: CRT: capillary refill time, SEC: seconds, CF: cardiac frecuency, BPM: beats per minute, RF: respiratory frecuence, BPM: breaths per minute, AAP: average arterial preasure, T: corporal temperature, M/S/P: moist, shiny pink, DP: dry and pink, PM: pale and moist, NA: not analyzed, NBS: normal bowel sounds, IBS: increase bowel sounds, LQ: left quadrant, URQ: upper right quadrant, ULQ: upper left quadrant, LHL: left hind limb. Table S4. Venous gases results in all three horses using the Epoc Blood Analysis® equipment. Ad: plasma administration. Horse three is missing the measure during plasma administration. Raw 12% SDS-PAGE electrophoresis results of albumin batch AL-15062023 at different concentrations. (DOCX) [file pone.0341577.s001.docx]

Article

Evaluation of the clinical response after intravenous admin-istration of equine albumin in adult Colombian Creole horses (*Equus ferus caballus*).

**Supplemetary material.**


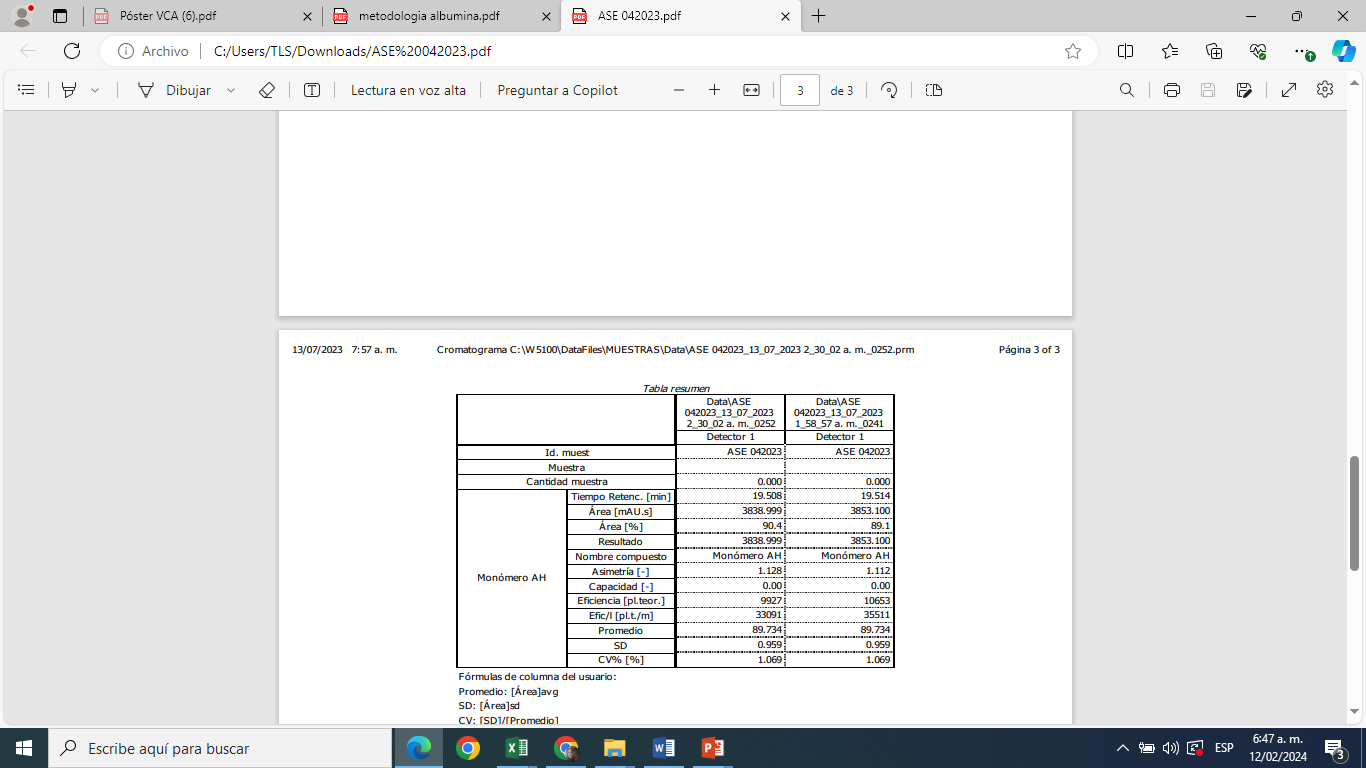


**Table S1.** Original result data tablesheet (in spanish) obtained from the SEC chromatography software.

**Table S2.** Transcribed and translated data sheet results table obtained from the SEC chromatography software (the original is available in the supplementary material). Highlighted data indicates the percentage of relative abundance of the fraction corresponding to human albumin (AH).

**Figures S1.** Original sterility assay result issued by the GLP certified laboratory (in spanish) indicating the sterility of **A:** Batch AL-15052023. **B:** Batch AL-02042024. **C:** Batch AL-10042024. English translated analysis certificates **D:** Batch AL-15052023. **E:** AL-02042024. **F:** AL-10042024.

**A**

**B**

**Figures S2.** Hemotropic assay result issued by the GLP certified laboratory indicating the absence of *Anaplasma sp*, *Babesia sp*, *Mycoplasma sp*, *Trypanosoma sp* and *Theileria sp*. on batch AL-15052023. **A:** Original report analysis in spanish. **B:** English Translation.

| **Horse 1** | | | | | | | | | | | | |
| --- | --- | --- | --- | --- | --- | --- | --- | --- | --- | --- | --- | --- |
| **Time (min)** | **Mental status** | **T. Skin fold** | **Mucous membrane color** | **CRT (SEC)** | **CF (BPM)** | **RF (BPM)** | **AAP (Hg mm)** | **Sistolic preasure (Hg mm)** | **Diastolic preasure (Hg mm)** | **T (C°)** | **Digestive auscultation** | **Clinical notes** |
| 0 min | Alert | 2 | M/S/P | 2 | 52 | 20 | 77 | 119 | 65 | 38,3 | NBS | None |
| 22 mins | Alert | 1 | M/S/P | 2 | 68 | 32 | NA | NA | NA | 38,4 | IBS - LQ | Positive pulse LHL |
| 60 mins | Alert | 2 | M/S/P | 2 | 125 | 40 | 86 | 141 | 65 | 38,6 | NBS | Positive pulse LHL |
| 80 mins | Alert | 2 | M/S/P | 2 | 94 | 40 | 73 | 124 | 51 | 38,6 | IBS - URQ | Positive pulse LHL |
| 240 mins | Alert | 2 | M/S/P | 2 | 75 | 44 | NA | NE | NA | 38,7 | NBS | None |
| 480 mins | Alert | 2 | M/S/P | 2 | 57 | 32 | 74 | 110 | 63 | 37,1 | NBS | None |
| 1440 mins | Alert | 2 | M/S/P | 2 | 80 | 40 | NA | NA | NA | 38,5 | IBS | None |
| **Horse 2** | | | | | | | | | | | | |
| **Time (min)** | **Mental status** | **T. Skin fold** | **Mucous membrane color** | **CRT (SEC)** | **CF (BPM)** | **RF (BPM)** | **AAP (Hg mm)** | **Sistolic preasure (Hg mm)** | **Diastolic preasure (Hg mm)** | **T (C°)** | **Digestive auscultation** | **Clinical notes** |
| 0 | Alert | 1 | M/S/P | 1 | 52 |  | 64 | 93 | 55 | 37,1 | IBS | Positive pulse |
| 30 | Alert | 1 | DP | 3 | 57 |  | 76 | 129 | 59 | 37,6 | IBS - LQ | None |
| 60 | Alert | 1 | PM | 2 | 53 | 24 | 77 | 117 | 68 | 37,7 | IBS | None |
| 180 | Alert | 1 | PM | 3 | 50 | 24 | 108 | 160 | 91 | 37,7 | IBS - LQ | None |
| 240 | Alert | 1 | PM | 2 | 52 | 36 | NA | NA | NA | 37,7 | NBS | None |
| 1440 | Alert | 1 | M/S/P | 2 | 52 | 40 | 71 | 116 | 61 | 38 | IBS - URQ | Whinny |
| **Horse 3** | | | | | | | | | | | | |
| **Time (min)** | **Mental status** | **T. Skin fold** | **Mucous membrane color** | **CRT (SEC)** | **CF (BPM)** | **RF (BPM)** | **AAP (Hg mm)** | **Sistolic preasure (Hg mm)** | **Diastolic preasure (Hg mm)** | **T (C°)** | **Digestive auscultation** | **Clinical notes** |
| 0 | Alert | 1 | PM | 2 | 54 | NA | 84 | 112 | 77 | 37,6 | IBS | None |
| 10 | Alert (nervous) | 1 | PM | 3 | 70 | NA | 112 | 185 | 86 | 38,1 | IBS | Icteric sclera |
| 30 | Alert | 1 | PM | 3 | 49 | NA | 73 | 85 | 68 | 38,1 | IBS - ULQ | Slightly icteric sclera |
| 60 | Alert (nervous) | 1 | PM | 2 | 48 | NA | 82 | 133 | 68 | 38,1 | NBS | Slightly icteric sclera |
| 1440 | Alert (nervous) | 1 | PM | 2 | 60 | NA | NA | NA | NA | 38,1 | IBS - LQ | Slightly icteric sclera |

**Table S3.** Table results with the physiological constants including the arterial preasure measurment. Table abreviations: CRT: capillary refill time, SEC: seconds, CF: cardiac frecuency, BPM: beats per minute, RF: respiratory frecuence, BPM: breaths per minute, AAP: average arterial preasure, T: corporal temperature,  M/S/P: moist, shiny pink, DP: dry and pink, PM: pale and moist, NA: not analyzed, NBS: normal bowel sounds, IBS: increase bowel sounds, LQ: left quadrant, URQ: upper right quadrant, ULQ: upper left quadrant, LHL: left hind limb.

|  | **Horse 1** | | | **Horse 2** | | | **Horse 3** | |
| --- | --- | --- | --- | --- | --- | --- | --- | --- |
| **Analito** | **Before ad.** | **During ad.** | **After ad.** | **Before ad.** | **During ad.** | **After ad.** | **Before ad.** | **After ad.** |
| **pH** | 7,471 | 7,456 | 7,465 | 7,445 | 7,451 | 7,453 | 7,455 | 7,407 |
| **pCO2 (mmHg)** | 30,7 | 30,4 | 30,3 | 36,7 | 39,8 | 39,9 | 32,6 | 40,9 |
| **PO2 (mmHg)** | 32,4 | 39,5 | 38,1 | 50,3 | 45,8 | 38,6 | 38,6 | 28 |
| **pH(T) (mmHg)** | 7,452 | 7,435 | 7,438 | 7,443 | 7,44 | 7,442 | 7,446 | 7,391 |
| **pCO2 (T) (mmHg)** | 32,5 | 32,3 | 32,8 | 36,9 | 41,1 | 41,1 | 33,4 | 42,9 |
| **pO2(T) (mmHg)** | 35,5 | 43,6 | 43,2 | 50,7 | 48,2 | 40,5 | 40,3 | 30,3 |
| **Chco3- (mmol/L)** | 22,4 | 21,4 | 21,8 | 25,2 | 27,7 | 27,9 | 22,9 | 25,7 |
| **BE(ecf) (mmol/L)** | -1,2 | -2,5 | -1,9 | 1,1 | 3,8 | 3,9 | -1 | 1 |
| **Cso2 (%)** | 67,8 | 77,9 | 76,6 | 86,9 | 83,5 | 75,6 | 76,5 | 53,1 |
| **Na (mmol/L)** | 135 | 136 | 137 | 136 | 136 | 134 | 132 | 136 |
| **K (mmol/L)** | 3,9 | 2,2 | 3,9 | 4,2 | 3 | 3,1 | 4,4 | 2,9 |
| **Ca (mmol/L)** | 1,47 | 1,35 | 1,44 | 1,45 | 1,43 | 1,56 | 1,54 | 1,43 |
| **Cl (mmol/L)** | 105 | 108 | 107 | 105 | 102 | 102 | 104 | 106 |
| **GAP (mmol/L)** | 9 | 8 | 10 | 7 | 7 | 5 | 7 | 5 |
| **Agapk (mmol/L)** | 13 | 10 | 14 | 11 | 10 | 8 | 11 | 8 |
| **HTO (%)** | 32 | 28 | 35 | 39 | 30 | 30 | 29 | 25 |
| **cHgb (g/dL)** | 10,7 | 9,4 | 12 | 13,1 | 10,3 | 10,2 | 9,8 | 8,4 |
| **BE (mmol/L)** | -0,6 | -1,9 | -1,1 | 1,3 | 3,5 | 3,7 | 0,6 | 0,9 |
| **Glu (mg/Dl)** | 99 | 111 | 112 | 86 | 94 | 113 | 107 | 153 |
| **Lac (mmol/L)** | 1,24 | 0,93 | 1,42 | 4,22 | 1,01 | 1,26 | 0,77 | 0,7 |
| **BUN (mg/dL)** | 17 | 21 | 20 | 13 | 14 | 13 | 9 | 8 |
| **Urea (mmol/L)** | 6,2 | 7,5 | 7 | 4,8 | 4,9 | 4,7 | 3,4 | 3 |
| **Crea (mg/dL)** | 1,3 | 1,43 | 1,55 | 1,17 | 1,11 | 1,18 | 1,17 | 1,26 |

**Table S4.** Venous gases results in all three horses using the Epoc Blood Analysis® equipment. Ad: plasma administration. Horse three is missing the measure during plasma adminisitration.


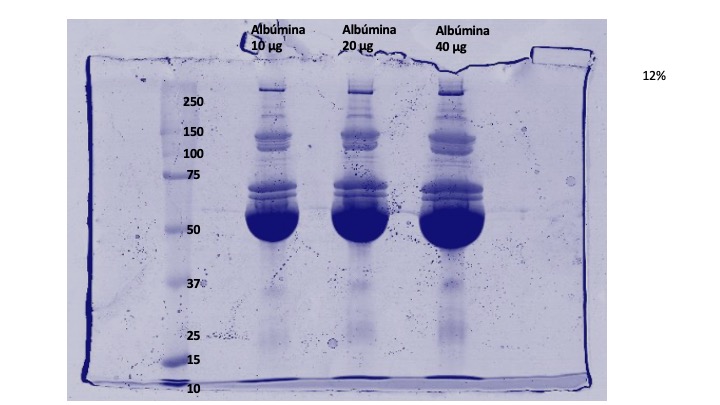


Raw 12% SDS-PAGE electrophoresis results of albumin batch AL-15062023.
